# Natural warning signals unexpectedly shape human metamemory ratings but not image recognition success

**DOI:** 10.1038/s41598-026-41178-y

**Published:** 2026-02-25

**Authors:** Federico De Filippi, Olivier Penacchio, Akira R. O’Connor, Julie M. Harris

**Affiliations:** 1 School of Psychology & Neuroscience, University of St Andrews, St Andrews, KY16 9JP UK; 2 Bridging Research in AI and Neuroscience, Computer Vision Center, Cerdanyola del Vallès, 08193 Barcelona, Spain; 3 Computer Science Department, Universitat Autònoma de Barcelona, Cerdanyola del Vallès, 08193 Barcelona, Spain

**Keywords:** Aposematism, Warning signal, Metamemory, Memorability, Receiver psychology, Vision, Ecology, Ecology, Neuroscience, Psychology, Psychology, Zoology

## Abstract

Although the bold warning signals of prey (known as aposematic) have been shown to facilitate predator learning through repeated encounters, it remains unclear to what extent their visual patterns and colours support memory retention. Here, we tested whether aposematic species appear more memorable to human observers, and whether they have an intrinsic advantage in being recognised from memory—even after a single exposure. Observers viewed images of aposematic and non-aposematic butterflies and moths, judged how likely they were to remember each one (metamemory rating), and later completed a test distinguishing previously seen species from novel ones (recognition memory). While aposematic patterns elicited higher metamemory ratings upon first sight, we found no evidence that they were more likely to be recognised when seen again. Despite this apparent metacognitive failure, for aposematic species the observers tend to remember and forget the same images as one another. This suggests that these images exhibit ‘memorability’, an intrinsic property of an image that allows one to predict how well images can be remembered. These findings raise the possibility that an effective visual warning may hinge less on recognition of a previously seen signal, and more on perceptual processes at play when it is first encountered.

**Supplementary Information:**

The online version contains supplementary material available at 10.1038/s41598-026-41178-y.

## Introduction

When we glance at an image, it can leave a lasting trace in memory, making it easy to recognise when encountered again^1,2^. This apparently effortless encoding of visual information is not only a hallmark of human memory but may also have an evolutionary significance in nature. One adaptation thought to exploit these processes is aposematism—the use of honest ‘warning signals’ of prey toxicity or unpalatability^3,4^ as a defence against predation. How the visual components of such signals, including their colours and patterns, influence predators’ foraging decisions is still not fully understood^5^.

The effectiveness of a warning signal pattern is commonly attributed to the ease with which it is remembered by predators^6,7^. Conspicuous patterns combining red, orange, and yellow with black, typically in striped or spotted arrangements, are successful aposematic signals, reliably avoided by predators in both natural and experimental contexts^8–13^. Most studies have focussed on how the conspicuousness of a signal promotes avoidance when predators are repeatedly exposed to it. To the best of our knowledge, there have been no attempts to directly compare how aposematic and non-aposematic species are recognized from memory on a purely visual level.

Here, we drew on well-known properties of human visual memory for images^14–16^ to test whether images of Lepidoptera (butterfly and moth species) that display warning signal patterns are easier to remember than those that do not. A growing trend in sensory ecology leverages human vision science to understand how animal patterning influences the perceptual mechanisms of the observer (reviewed in^17^)*.* There is evidence that the effectiveness of warning signal patterns is rooted in early vision mechanisms, such as edge detectors^18^ and colour opponency processes^19^, which are broadly shared by humans and birds^20,21^, arguably the primary predators of Lepidoptera^13^. Researchers have used human observers to test what disrupts or enhances the detectability of prey targets, revealing key principles of camouflage and conspicuousness^22–29^. This comparative approach allows traits that have proved adaptive in nature to be characterised in detail through measuring human visual behaviour. The ability to be remembered is considered crucial for effective animal signalling^6,7^ and can be easily measured in humans. However, humans have not yet been used as experimental observers to assess whether warning signals provide an advantage in recognising prey from memory. Accordingly, this study targets the intersection of visual cognition and sensory ecology, using human behaviour to address questions motivated by predator–prey signalling.

In prior ecological work, our group constructed a database of hyperspectral images of aposematic and non-aposematic Lepidoptera and used computational neuroscience methods to characterise how coloration patterns may stimulate the brain of predator birds^30^. The modelling demonstrates that the patterns of aposematic species evoke different neural responses than those from non-aposematic ones, providing a framework to quantify the role of visual warning signal patterns. Here, building on this work, we asked whether these same warning signal patterns might also influence human memory. We selected species from the database predicted to evoke the strongest and weakest responses in the avian visual pathway and measured several aspects of human memory in response to these images, defined below.

1. *Metamemory:* Observers can form intuitions about how likely they are to remember a stimulus in the future, measured via metamemory ratings^31,32^. Such ratings can be accurate when based on specific cues (e.g., whether images ‘tell a story’^33^), but are often unreliable when observers lack these cues^14^. Relatedly, warning-coloured stimuli for predator avoidance experiments are often chosen according to how conspicuous or distinctive they appear to human observers.
2. *Recognition memory*: Humans can store detailed representations of thousands of images in memory, even after seeing them only once^1,2^. Recognising a previously seen image can be thought of as being effortless (^1,2^, though see also^34^). This ability makes humans a useful model for testing whether aposematic signals exploit memory mechanisms.
3. *Memorability*: Recognition success varies systematically across images—some are reliably recognised by most people, but others only by a few. Whether one person recognises an image can be predicted from whether others do^14^, indicating that recognition is not entirely idiosyncratic, but partly image-specific^35^. Memorability is thus defined as an intrinsic, predictable property of images^15,16,36^. Neuroimaging^15^ and electrophysiology^37^ suggest that it has a perceptual basis, related to how the visual system responds to images, and it can also be predicted computationally from image content alone^14,38,39^. This framework allows us to test whether aposematic species possess visual features that intrinsically influence predator decisions, revealing a previously unexplored dimension of their adaptive function.

We tested whether aposematic species would (1) be rated by participants as easier to remember (metamemory rating), (2) be better remembered in a recognition task, (3) exhibit an association between metamemory ratings and recognition performance, and (4) promote consistent memory performance across individuals (memorability). Our aim was to assess whether visual features considered adaptive in ecological contexts truly enhance these aspects of memory.

## Results

Human observers viewed images of aposematic (AP) and non-aposematic (non-AP) Lepidoptera species and provided subjective estimates of how likely it was they would remember each species if encountered again (i.e., metamemory rating). Later, observers completed a recognition memory test, where they viewed a mix of previously seen images (i.e., ‘targets’) and novel ones (i.e., ‘lures’) and had to decide whether each image was ‘old’ or ‘new’ (i.e., recognition judgement). Images were selected a priori to maximally differ in how they stimulate the luminance and colour pathways of a model avian predator visual system (our previous work^30^ showed that this model can effectively separate AP and non-AP species into separate categories based on that specific stimulation). For details on the experimental task and selection of visual stimuli, see *Materials and Methods*. We compared the effects of AP and non-AP species on observers’ metamemory ratings and recognition. Subsequently, we analysed the consistency of recognition across observers, to estimate the amount of intrinsic memorability delivered by AP and non-AP species.

### Metamemory rating: do aposematic species appear more memorable?

**Fig. 1:**
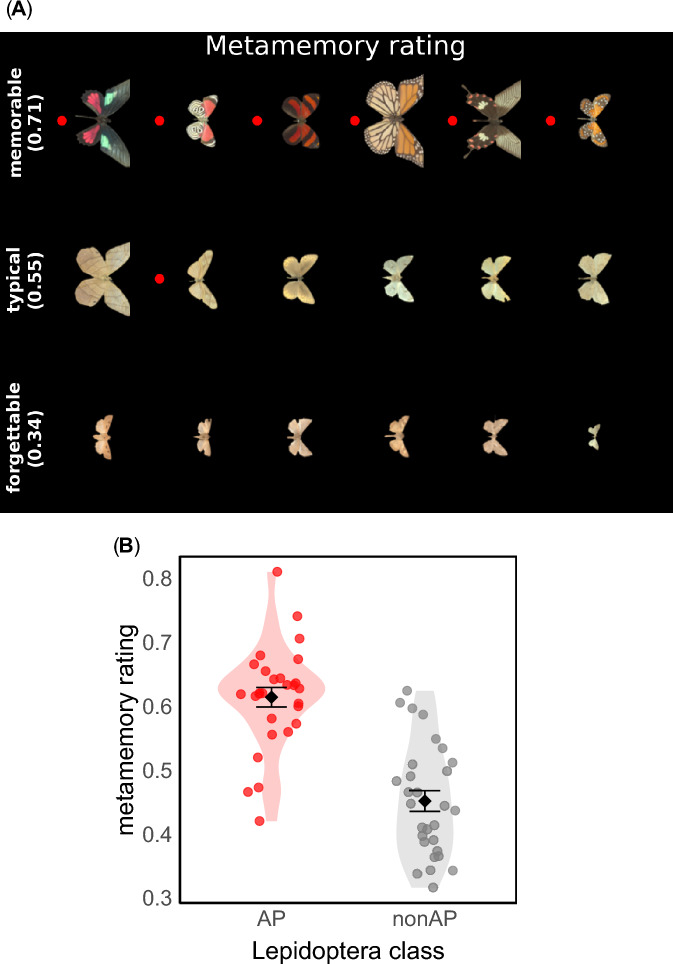
(**A**) Examples of species from the St Andrews Hyperspectral Lepidoptera Database (https://arts.st-andrews.ac.uk/lepidoptera/index.html), sorted by the average metamemory rating given by human observers (0 = ‘very forgettable’, 1 = ‘very memorable’). The red dots mark aposematic (AP) species. The percentage is the average metamemory rating for that row of images. The ‘memorable’ and ‘forgettable’ rows show the images with the highest and lowest mean metamemory rating. The ‘typical’ row shows the images with the smallest differences from the median of scores. The mean metamemory rating for each species was calculated by averaging ratings across the whole subject pool. (**B**) Metamemory rating for each image, grouped by whether species were aposematic (AP, red dots) or not (non-AP, grey dots). Each point shows the mean rating of one image across the subject pool. Points are randomly jittered along the x-axis. Error bars display the mean rating ± 1 standard error of the mean.

When each target species was viewed for the first time, observers provided a metamemory rating, a score describing the perceived likelihood of remembering the image when encountered in the future (see *Materials and Methods—Metamemory rating block*). Figure 1A shows examples of species that were rated as most memorable, typical, and forgettable. Figure 1B shows the average metamemory rating for each image, grouped by whether items were AP or non-AP.

We fitted a linear mixed-effects model predicting metamemory ratings from Lepidoptera class (AP vs. non-AP), including both random intercepts and slopes for participants [[rating ~ class + (class | participant)]]. There was a significant effect of class on metamemory (β = 0.16, SE = 0.02, t = 8.75, *p* < 0.001; Fig. 1B), indicating on average, AP species (mean = 0.61, SE = 0.02) were rated 0.16 units higher than non-AP species (mean = 0.45, SE = 0.02). Random effect estimates showed individual differences in ratings: the standard deviation of the participant intercepts was 0.16, and the standard deviation of the Lepidoptera class slopes was 0.12. This indicates that most observers rated AP species between approximately 0.04 and 0.28 units higher than non-AP species. The proportion of variance in metamemory ratings explained by the fixed effect (marginal R^2^) was 10.8%. The proportion of variance explained by fixed plus random effects (conditional R^2^), was 47.5%. These results confirm that, despite individual differences, on average, AP species were perceived as more likely to be remembered than non-AP species upon first sight.

### Recognition: are aposematic species easier to remember?

**Fig. 2:**
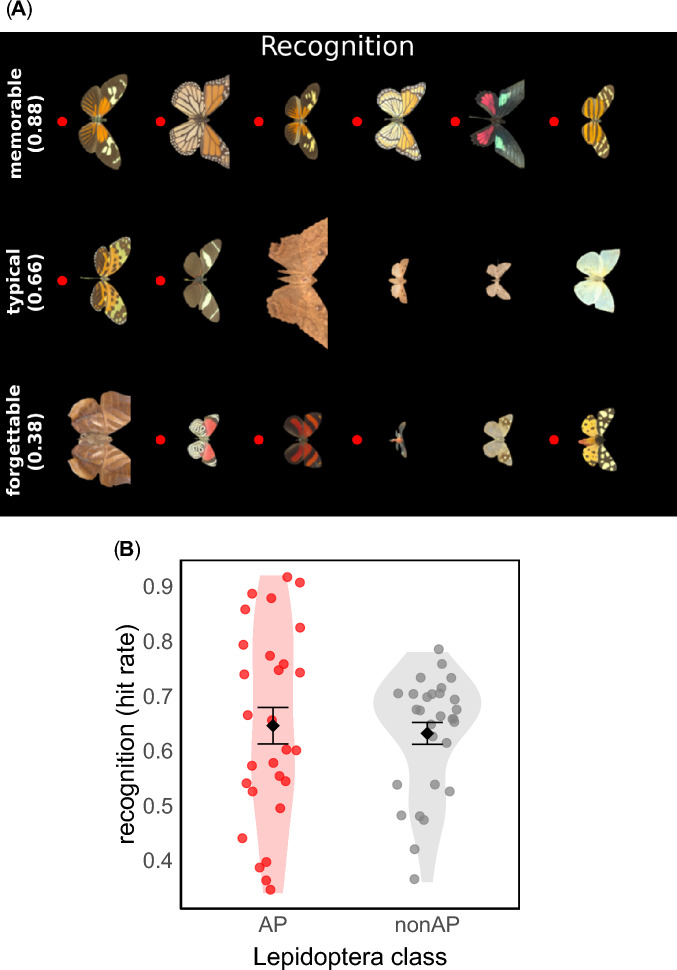
(**A**) Examples of species from the St Andrews Hyperspectral Lepidoptera Database, sorted by the proportion of correct recognitions (i.e., hit rate) they elicited across the subject pool. Red dots mark aposematic (AP) species. The percentage for each row shows the mean recognition hit rate for that row of images. The ‘memorable’ and ‘forgettable’ rows show the images with the highest and lowest mean hit rates. The ‘typical’ row shows the images with the smallest differences from the median hit rate. (**B**) Recognition hit rate for each image, grouped by whether species were aposematic (AP, red dots) or not (non-AP, grey dots). Each point shows the mean rating of one image across the subject pool. Points are randomly jittered along the x-axis. Error bars display the mean rating ± 1 standard error.

We next measured whether images were recognised when viewed again. In an old/new recognition test, a previously seen target is either successfully recognised as familiar (i.e., ‘hit’) or not recognised (i.e., ‘miss’); similarly, a lure can either be successfully recognised as novel (i.e., ‘correct rejection’) or mistakenly judged as familiar (i.e., ‘false alarm’). To evaluate recognition performance, we focused on hits (but see *Supplementary Information—Complementary measures of recognition* for additional analyses that address response bias). Figure 2A shows examples of species sorted by the hit rate. Figure 2B shows the average hit rate for each image, grouped by whether items were AP or non-AP.

We fitted a logistic mixed-effects model predicting memory outcome (hit vs. miss) from Lepidoptera class (AP vs. non-AP), including both random intercepts and slopes for participants [[recognition ~ class + (class | participant)]]. There was no significant effect of Lepidoptera class on recognition outcome (β = 0.06, SE = 0.13, z = 0.45, *p* = 0.651; Fig. 2B), suggesting that AP species (mean = 0.65, SE = 0.03) were not more likely to be recognised than non-AP species (mean = 0.63, SE = 0.02). The proportion of variance in recognition outcomes explained by the fixed effect alone was close to zero (0.02%), while the variance explained by the full model including random effects was 10.84%. These results do not provide support for the idea that recognition memory is better for AP species than non-AP species (see Fig. 2).

### Comparing recognition and metamemory ratings: is memory for Lepidoptera intuitive?

**Fig. 3:**
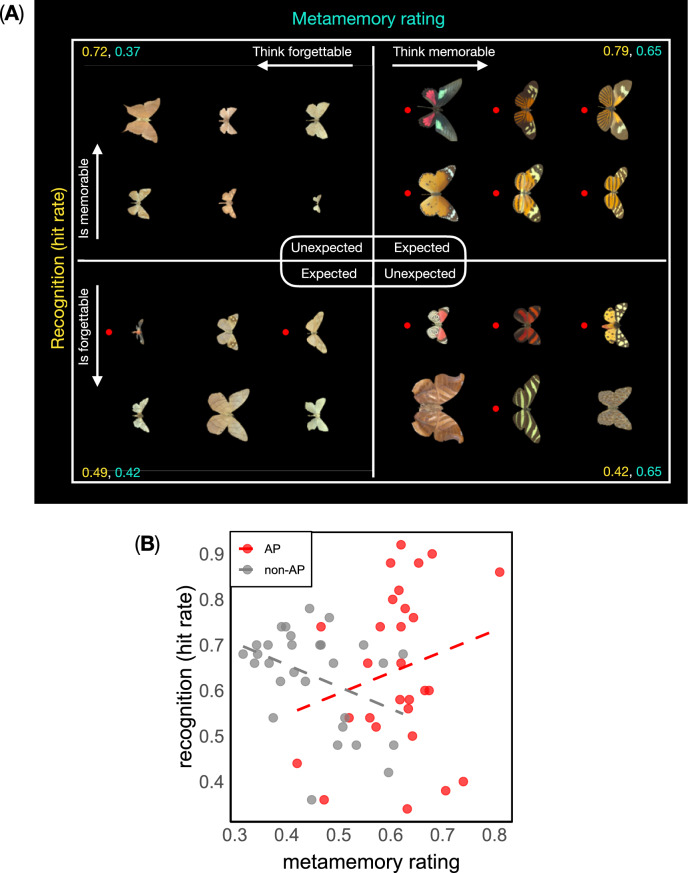
(**A**) Examples of the strongest matches (‘expected’) and mismatches (‘unexpected’) between observer’s perception of how memorable each species appeared when seen for the first time (metamemory rating), and recognition success (hit rate). The red dot highlights aposematic (AP) species. For example, species from the top-right set were firstly rated as highly memorable and subsequently recognized by 79% of observers. In contrast, species from the bottom-right set were also rated as highly memorable but recognised only by 42% of observers. (**B**) scatterplot and best linear fits of the correlation between recognition and metamemory ratings, grouped by Lepidoptera class.

Can we predict whether species would be remembered or forgotten in the future simply based on how ‘memorable’ they appear to us at first exposure? The answer is hinted at by the evident discrepancy between the observed metamemory and memory results (see Figs. 1 and 2). Here we provide a more detailed analysis. We calculated the difference between metamemory ratings and recognition hit rates for each image. We then plotted the species with the smallest (‘expected’: strongest matches between the two measures) and largest (‘unexpected’: weakest matches) differences, shown in Fig. 3A. Subsequently, we calculated the correlation between the metamemory scores of each image and recognition hit rates (Fig. 3B). Overall, metamemory and recognition across both classes were uncorrelated (Spearman’s ρ = 0.01, *p* = 0.930). There was a non-significant correlation for AP species (ρ = 0.21, *p* = 0.280; Fig. 3B), and there was a small but significant negative correlation for non-AP species (ρ = −0.39, *p* = 0.037; Fig. 3B).

Given the observed dissociation between metamemory ratings and hit rates, we conducted additional analyses examining two image properties that might explain variation in these measures: overall specimen size, and visual statistics (luminance, colour, edges) of the patterns (as per our model^30^). These results are reported in the *Supplementary Information*. Specimen size influenced metamemory ratings for both AP and non-AP species, but it influenced recognition performance only for AP species (Supplementary Figure S4). Visual statistics influenced meta-memory for both AP and non-AP species but did not influence recognition for either class (Supplementary Figure S6).

### Memorability: is memory for Lepidoptera predictable across observers?

Having found clear differences in metamemory between AP and non-AP species, but not in memory (as measured by recognition), we next asked whether warning signal patterns might influence recognition in ways not captured by overall performance measures. To assess this point, it is necessary to quantify individual differences using a ‘consistency analysis’^14^, a standard method in memorability research for evaluating if memory is predictable across observers. The consistency analysis involves repeatedly splitting the full subject pool into two random halves (which we label group j and group k), calculating hit rates per image separately within each group, and assessing consistency in hit rates across groups using two complementary metrics.

**Fig. 4:**
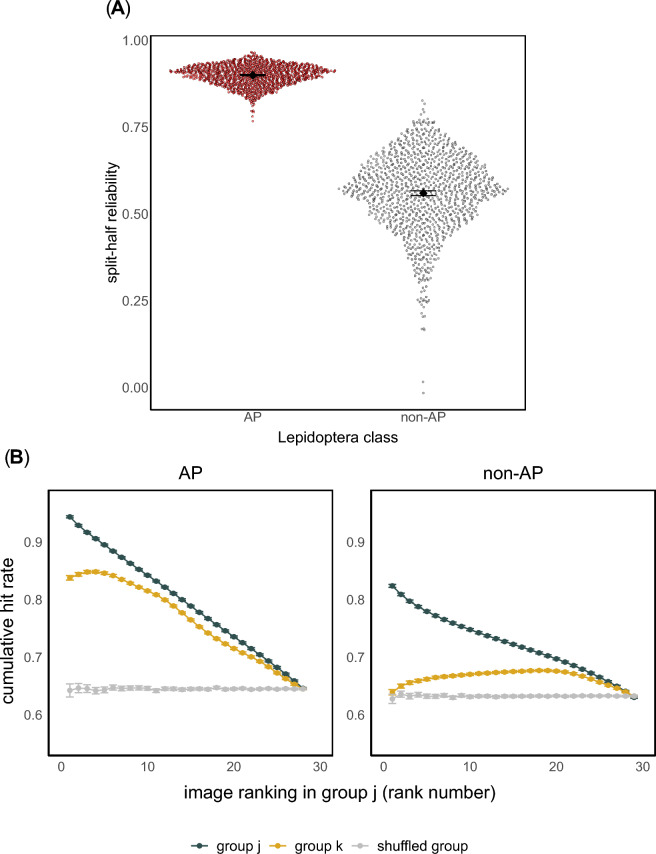
Visualization of the consistency analysis used to estimate the intrinsic memorability of aposematic (AP) and non-aposematic (non-AP) Lepidoptera. (**A**) Split-half reliability (i.e., Spearman-Brown corrected correlation coefficient) calculated from 1000 random splits of subjects, grouped by Lepidoptera class. Each point shows the split-half reliability between two random splits from one iteration of the consistency analysis. Error bars show the mean split-half reliability ± bootstrapped 95% confidence intervals for each class. (**B**) Memory consistency across observers as a function of the hit rate of images. Hit rates for each image were sorted in descending order according to one group (x-axis) and plotted against the cumulative average of the hit rates (y-axis) in another group, or in a randomly shuffled distribution of hit rates. When group j and group k curve overlap and differ the most from the shuffled group curve, consistency between observers is high. Each point is the average hit rate across all 1000 iterations of the analysis. Error bars show means ± bootstrapped 95% confidence intervals of the cumulative hit rates across all iterations.

The first is split-half reliability, a metric which indicates how stable hit rates are across different observers, calculated as the Spearman-Brown corrected correlation between hit rates from each group j and k. We calculated an overall split-half reliability of ρ = 0.80 (95% CI [0.80, 0.81]) across all Lepidoptera species. Next, we calculated it separately for AP and non-AP species. The average split-half reliability for AP species was ρ = 0.90 (95% CI [0.90, 0.90]) and for non-AP species it was ρ = 0.56 (95% CI [0.56, 0.55]). This is shown in Fig. 4A. Note that split-half reliability quantifies consistency in rank-ordering of hit rates between groups but is independent of overall recognition accuracy. Correlations can be high whether participants tend to remember or forget the same images^40^. Conversely, low split-half reliability suggests that participants disagreed about which images were remembered or forgotten. The difference in split-half reliability between AP and non-AP species was statistically significant (Δρ = 0.35, permutation test, *p* < 0.001; Fig. 4A; see *Materials & Methods—Statistical testing of consistency measures*).

The second metric, shown in Fig. 4B, uses cumulative hit rates across the different subject splits^14^ to estimate how well performance from group j predicts that of group k, providing a more detailed breakdown of consistency as a function of recognition accuracy. If particular images are especially likely to be remembered (or forgotten) by one group, will a different group show the same pattern? To answer this question, image-wise hit rates were sorted in descending order (i.e., from most memorable to forgettable) according to group j and compared against those from group k, or against a baseline shuffled group, created by randomly permuting the association between images and their hit rates in group k, while keeping the image ordering for group j fixed. The curves in Fig. 4B were constructed by calculating the cumulative hit rate (mean hit rate of top-n images) for each group, while moving across this fixed image ordering (i.e., from the top-2 to the top-28 most remembered images), for each of the 1000 random subject splits shown in Fig. 4A. High consistency would yield a close match between the curves for group j and group k, and a separation from the shuffled group curve. This can be interpreted as meaning that different individuals remembered and forgot the same images in a systematic, non-random way. Conversely, low consistency would yield greater separation between group j and group k curves, and a closer match with the shuffled group. For example, Fig. 4B shows that the top three most-remembered AP images were recognised by approximately 95% of observers in one group and by 85% in another, whereas the top three non-AP images were recognised approximately by 80% and 65%, respectively.

We calculated the difference in cumulative hit rates between groups at each image rank (see *Materials & Methods—Statistical testing of consistency measures*). For AP species, we found significant differences between group k and the shuffled group at all image ranks except the last rank (Bonferroni-corrected *p* < 0.001 for all ranks, *p* = 1.000 for rank 28; Fig. 4B). For non-AP species, group k significantly differed from the shuffled group at all ranks except the first and last rank (*p* = 1.000) and marginally at rank 2 (*p* = 0.057; all other ranks *p* < 0.001; Fig. 4B). Between classes, AP and non-AP species differed significantly in how much the cumulative hit rate of group k exceeded its shuffled baseline (*p* < 0.001 at all ranks except rank 26, *p* = 1.000). This is shown in Fig. 4B, where the group k and j curves for AP species remained closely aligned across most ranks, whereas non-AP species showed weaker alignment, especially for the most memorable images.

## Discussion

This study tested whether the warning signal patterns of aposematic (AP) Lepidoptera species enhance visual memory in human observers. Our measurement of metamemory rating showed that AP species appear to participants as though they will be more memorable than non-AP species. Surprisingly, this was not backed up by actual memory performance: recognition was not better for AP than for non-AP species. Yet, our analysis showed that people do tend to remember the same images as one another, and their recognition performance is more similar to that of others when the species carry AP patterns. In the following, we discuss how these results fit within the current literature and offer a novel contribution to understanding the cognitive processes underlying visual warning signals.

In previous research, we used computational modelling of both the colour and luminance channels involved in visual processing to show that the AP species used in this study evoke stronger activity in visual brain areas than the non-AP species^30^. Here, the AP species were the ones that our participants chose as most likely to be remembered. This result did not surprise us. It is reminiscent of the long-standing idea, dating back to Darwin and Poulton^3^ and still reiterated today^4^, that warning signal patterns appear particularly ‘striking’ to the human eye. However, our metamemory impression proved sometimes inaccurate, leading to both expected and unexpected outcomes (see Fig. 3). Among the ‘expected’ cases, AP species with visually distinctive markings and colours were judged memorable and later recognised (‘memorable–expected’), whereas non-AP species that seemingly lack warning colouration were judged most forgettable and later forgotten (‘forgettable–expected’). By contrast, the most ‘unexpected’ species showed less obvious visual distinctions between recognised and forgotten images.

We do not know why visually distinctive patterns drive us to think they will be easily remembered. Our results do concur with some related human memory literature on complex visual scenes. Observers can be notoriously poor at anticipating which scene photographs will later be remembered^14^, with metamemory measures showing little correspondence with real recognition performance, as we found in this study. By contrast, metamemory ratings can be accurate when they are based on specific semantic attributes of photographs (e.g., peacefulness, presence of people^33^), which were minimised in our stimuli depicting single specimens. Our data support the notion that naturalistic metamemory judgements sometimes do not reflect memory outcomes. Rather, bringing us back to the idea that warning signals are ‘striking’^3^, metamemory ratings have been shown to predict ratings of image interestingness and aesthetics more successfully than recognition performance^14^. We speculate that whatever strikes us as potentially ‘memorable’ about some patterns or photographs is at least partly driven by systems closely linked to vision^15^, rather than solely by our memory systems.

Our findings do suggest that warning signal patterns are doing something more subtle than triggering the memory systems involved in recognition. Some earlier authors^5^ have emphasised a similar complexity, in understanding predator learning and decision making when faced with aposematic prey. In a comprehensive review, they highlight that avoidance learning (where a predator learns over time that a prey item is toxic or is otherwise not valuable to eat) is governed by a complex balance between gathering information about the prey (e.g., via conspicuous warning signals) and the cost of eating the prey (e.g., a build-up of toxicity level). If gathering information about a prey which has a strong AP pattern is easier, then learning will likely be faster, but this does not require the patterns to be specifically easier to *remember*. If the ‘striking’ perception of humans when faced with AP patterns that we observed in this study transfers to other predators, perception-led cognition, linked to the memorability of specific images, could drive learning, alongside or independent of traditional memory systems. Overall, our results suggest that a strong AP pattern is best characterised by how it appears at first exposure, rather than how easily it is recognised from memory later, shedding some light on what makes warning signal patterns effective.

Note that our study cannot fully exclude the possibility that warning signal patterns confer certain recognition advantages. As noted in *Materials and Methods*, our stimulus set necessarily included only AP species as lure images in the recognition task, which may have introduced response biases. However, confidence ratings (reported in *Supplementary Information—Complementary measures of recognition*) did not differ across AP and non-AP species, providing no evidence that such biases strongly influenced recognition. Overall, the balance of evidence in this study is more consistent with perceptual than with recognition-based effects of warning signal patterns.

In recent years, the concept of memorability, in particular applied to images, has been developed by the work of Bainbridge and others (see recent reviews^16,36^). There is evidence that images that are memorable across many observers evoke distinctive patterns of activity in visual brain areas when viewed^37^, even in observers who later forget them^15^, suggesting perceptual, memorability-specific mechanisms in the brain that are dissociable from memory systems^15^. One possible reflection of this dissociation in our data is the disconnect we found between metamemory ratings and recognition. Another established behavioural signature of memorability mechanisms comes from consistency in memory across observers, which we also observed. The key evidence used to define the notion of “intrinsic” memorability is that, when faced with many images, some are easy to remember, and some difficult, yet crucially, the same images tend to be remembered and forgotten by different individuals. We analysed our data with this concept in mind. When comparing consistency between AP and non-AP patterns, we found that people are more likely to remember or forget the same patterns from the AP class, making hit rates strongly predictable (see Fig. 4A). This is reflected in our results by a near-perfect alignment in recognition performance across individuals, both for remembered and forgotten AP species, across the entire spectrum of memory accuracy (see Fig. 4B). This alignment was less pronounced for non-AP species.

Consistency in memory has been reported across a growing range of human-centred images designed for mass audiences, such as complex photographs (ρ = 0.75^14^), data visualisations (including infographics and graphs, ρ = 0.83^41^), paintings (ρ = 0.53^42^), and dance moves (ρ = 0.51^43^). However, comparing measures of consistency should be done with caution. Our image sample size was necessarily small compared to most other studies (see Methods), which could potentially result in unreliability of correlations. With that caveat in mind, we did find stronger correlations here (ρ = 0.80 for all species combined) than in most other studies. A key point for us is that consistency was considerably higher for warning signal patterns (ρ = 0.90 for AP, ρ = 0.56 for non-AP). Unlike the other studies, our image classes contained ecologically adaptive traits, thus we provide a first example of memory consistency being modulated by such traits, as well as being amongst the highest measured in any study (to our knowledge). Such a pattern suggests that warning signals might engage the perceptual processes responsible for memorability, known to operate independently of traditional memory systems^15^.

Note that our images do not fully fit the standard memorability framework, at least in terms of the classes of images that have been studied so far. Recall that our two image classes were chosen not only on the basis that the species depicted were known to be aposematic, or not, but also that each member of the class provided a ‘good’ example of that class, as determined by our neural modelling of the population response of early visual processing (^30^, see Methods and Supplementary Information for model details and statistics). Early memorability work showed that basic pixel statistics (hue, saturation, intensity^14^) are not predictive of memorability, and that memorable vs. forgettable images matched on colour and spatial frequency do not evoke different responses in early visual areas^15^, leading to the view that low-level vision may not significantly influence memorability. By contrast, recent evidence shows that even phase-scrambled images without any semantic content carry intrinsic memorability^44^, although which specific low-level image properties modulate this effect remains an open question. The differences we found while using this approach suggest that future memorability studies may benefit from quantifying low-level image statistics in a more biologically inspired fashion.

Physical image properties linked to the magnitude of visual stimulation, such as size^45^ and contrast^46^, have been shown to facilitate recognition. In our stimulus set, we preserved the natural relative size variation of Lepidoptera species, which did influence metamemory ratings for both AP and non-AP species, although it facilitated recognition only for AP species (see *Supplementary Information*). Because specimen size was not experimentally controlled, this should be considered a limitation of our study. While size may have acted as an auxiliary cue for recognition (consistent with evidence that larger images are better remembered^45^), it is unlikely to explain the observed lack of recognition advantage for AP species.

Our findings do not appear to be consistent with a recent study showing that higher-contrast images (as measured by Root Mean Square (RMS) contrast) are better remembered^46^. However, in that study, contrast was manipulated at the level of individual images to examine its influence on recognition. In our study, we instead compared recognition across stimulus sets that naturally differ in contrast, with image selection aimed at maximising differences in visual stimulation and no image manipulation. Although many of the AP patterns used here would score highly on RMS contrast, we did not find that those AP patterns facilitated recognition, but we did observe higher memory consistency. In addition, our framework differs in approach from that previous study (and prior memorability literature) in an important respect. Our population modelling framework captures the spatial arrangement of patterns in a way that is not reflected by simpler pixel-based contrast measures such as the RMS contrast, which remain unchanged if a pattern is spatially scrambled^30^. One possible explanation is that increasing RMS contrast enhances overall visual stimulation, which can facilitate recognition, while the spatial organisation of contrast across a pattern (captured by our approach) may influence how images are encoded across observers. It is well established that higher-contrast images elicit stronger early visual responses^47^, and our measures extend this principle by accounting for additional aspects of pattern design that may further increase neural activity. To sum up, our data offer the first evidence that natural warning signal patterns could be processed by memorability mechanisms, although our image set and findings differ from current memorability studies.

In conclusion, this study suggests that the visual processing of certain animal patterns may influence the initial viewing of an image in ways that make it appear more ‘memorable’ to the human eye. The standard ‘receiver psychology’ view that warning signals are effective for animal predators partly because they are well remembered^6^ was put forward without the knowledge we have discussed above, of the subtle ways in which vision and memory interact. Our work has begun to unpack the idea that AP patterns could be ‘easily remembered’ to reveal which mechanisms contribute to the effectiveness of animal warning signal patterns.

## Materials and methods

### Participants

57 participants recruited via the Prolific (www.prolific.com^48^) participant pool took part in the study. 7 participants who failed one or more vigilance checks (see *Materials & Methods—Vigilance check*) were excluded from the data pool, leaving us with N = 50 (25 male, 25 female, mean age = 28.98, SD = 8.30). The Prolific reimbursement rate for taking part in the study was set to £9 per hour (median completion time = 27 min). Participant selection criteria were normal or corrected-to-normal vision and no colour blindness. Informed consent was obtained from participants before the study. Participants were informed that they would be asked to look at pictures of butterflies and moths and provide subjective judgements, but information about which species were AP was not shared. Prior to being made available on Prolific, the study was approved by the local research ethics panel (University Teaching and Research Ethics Committee—UTREC, School of Psychology & Neuroscience, University of St Andrews). The experimental procedures were performed in accordance with the British Psychological Society (BPS) Code of Human Research Ethics and the Declarations of Helsinki.

### Selection of stimuli

When selecting Lepidoptera species to serve as stimuli in the memory experiment, our specific aim was to compile two sets of images (AP and non-AP species) predicted to be represented differently within the visual system of avian predators^30^. All images implemented in the study were selected from the publicly available St Andrews Hyperspectral Lepidoptera database (https://arts.st-andrews.ac.uk/lepidoptera/documentation.html). For details on image acquisition, see Penacchio et al.^30^.

The full database contains images of 125 butterfly and moth species from 12 Lepidoptera families: 96 aposematic (AP) and 29 non-aposematic (non-AP), sampled from British and American museum collections. In our previous work^30^, these species were first classified as AP or non-AP according to standard criteria in the zoological literature, based on evidence for toxicity and documented rejection by predators. Subsequently, the photographs of these species were analysed using a predator vision-modelling pipeline to assess whether the zoological ‘ground truth’ classification of AP versus non-AP could be explained from visual pattern information alone. Our model could indeed do that. The modelling steps are summarised below.

The predator vision model pipeline (originally developed by Penacchio et al.^30^) includes an achromatic pathway and a chromatic one, designed to approximate established luminance and colour mechanisms known to operate in the avian brain (and broadly shared with the human visual system^20,21^). The luminance pathway comprises units (i.e., “neurons”) with realistic receptive fields that are sensitive to oriented edges across a range of different orientations and spatial scales, thereby capturing not only overall pattern contrast but also the spatial structure and organisation of patterns. The colour pathway comprises units with receptive fields that are sensitive to chromatic contrast across the image, encoding colour information through opponent channels capturing red–green, yellow–blue, and ultraviolet–blue; analyses focused on the red–green axis, which accounted for the primary differences between pattern classes.

In prior work^30^, these pathways were applied to the images of AP and non-AP species to quantify population-level neural responses to lepidopteran patterns. Three summary statistics of population activity were computed to characterise responses to each pattern: luminance contrast (achromatic response magnitude), colour contrast (chromatic response magnitude), and Orientation Distribution Deviation (ODD), a measure designed to capture deviations from the consistent statistics in the distribution of contrast edge orientations in natural scenes. Natural images typically exhibit non-uniform orientation distributions, with horizontal and vertical orientations over-represented. AP patterns systematically deviate from this structure, with striped patterns producing highly anisotropic responses and spotted patterns producing more isotropic responses. Together, these statistics (available in the St Andrews Hyperspectral Lepidoptera database) show that AP and non-AP species occupy distinct regions of a three-dimensional ‘pattern space’.

The three summary statistics, derived from the predator vision modelling described above and shown to effectively discriminate AP from non-AP species^30^, were used here to select 29 AP species predicted to produce the strongest visual responses, and 29 non-AP species predicted to produce the weakest ones. The values of these statistics for the selected species (corresponding to the location of each species in the predator-vision pattern space) are shown in Supplementary Figure S1. These 58 species served as targets for memorisation in the experiment (shown in Supplementary Figure S2), while the remaining species were used as lures (Supplementary Figure S3). Given the small number of non-AP species in the original database, we used only AP species as lures and always presented non-AP species as targets. Note that an a-priori selection for targets and lures is unlike standard memory paradigms, where targets and lures are typically randomised across participants. Instead, our approach ensured that the two classes of targets were always maximally differentiated in their predicted neural representations, enabling a more direct test of whether these ecologically meaningful differences in visual encoding translate into differences in human memory.

### Presentation of stimuli

The selected species from the database were subsequently converted from hyperspectral images to the standard RGB colour space and presented in PsychoPy^49^. For details on the conversion of hyperspectral images see *Supplementary Information*—*Conversion of hyperspectral images to sRGB colour space*. This resulted in a database of pictures of single specimens of various sizes. For presentation, the converted images were resized to a resolution of 256 × 256 using bilinear interpolation and a constant scaling factor of 256/k, where k is the size of the largest image in the database. Given the evidence of common frequency components in Lepidopteran wing patterns^30^, this was done to preserve the natural size differences between specimens. The size of images in the online experiment was not controlled, but stimuli were set to occupy half of participants’ display and to be invariant to changes in aspect ratio of the browser window. Participants were asked to sit naturally in front of their computer, as they would in everyday settings. It is known that people sit at an average distance of approximately 60 cm in naturalistic viewing conditions^50^. We can estimate the approximate size as viewed by an average person sitting at an average viewing distance (D ≈ 60 cm), using an average 15-inch laptop display. Under these conditions, the smallest specimen would subtend approximately 0.39° of visual angle, whereas the largest specimen would subtend approximately 2.53°.

## Procedure

### Metamemory rating block

**Fig. 5:**
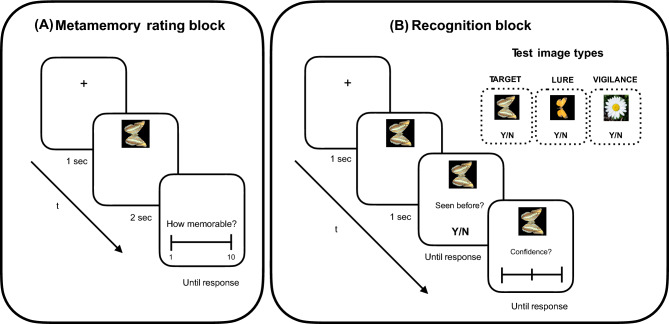
Examples of one trial from the metamemory and recognition blocks. (**A**) In the metamemory block, participants viewed all target images and rated each one based on how likely they thought they would remember it later. (**B**) In the recognition block, participants were shown a mix of previously seen images (‘targets’), novel ones (‘lures’), as well as task-irrelevant vigilance-check images, and had to decide whether each stimulus was ‘old’ (seen before) or ‘new’ (not seen before).

The experiment started with a ‘metamemory rating’ study block, followed by a recognition test block (Fig. 5). In the metamemory block (Fig. 5A), participants viewed all aposematic and non-aposematic targets and provided metamemory ratings. Participants were instructed to look at each image carefully and provide their subjective impression of how memorable it looked. The metamemory rating was explained to participants in the instructions as follows: ‘*A memorable image is one that you feel you would be likely to pick out as having seen before, if you saw it again soon, so you would give it a high score’.* At the start of each study trial, a central fixation marker appeared for 1 s, followed by one image that remained on screen for 2 s. Subsequently, the image disappeared, and the question ‘*How memorable is this image?*’ appeared on screen, along with a 10-point clickable rating scale. Participants were instructed to select a score that best described their subjective impression of how memorable the stimulus looked, from 1 (‘very forgettable’) to 10 (‘very memorable’). Each target was studied and rated only once, and the order of presented targets was randomised for each subject. Note that previous work that has compared memory and metamemory for photographs^14^ used binary memorable/forgettable ratings for scenes from many different classes (e.g., landscapes, urban, persons). Given that our targets were all of one image-type (i.e., different butterfly/moth species), we chose to implement a broader rating scale to measure finer-grained, within-class differences. At the end of the study block, a break was offered before the test block.

### Recognition block

In the ‘recognition’ test block (Fig. 5B), participants were instructed to look at images and state whether they had previously seen each one. Each test trial started with a 1-s central fixation marker, followed by one test image that remained on screen until the end of the trial. Unbeknownst to participants, the test image could be either a target (i.e., previously studied image), a lure (i.e., never seen before image) or a vigilance check (i.e., a task-irrelevant picture of a flower). After 1 s, the question ‘*Have you seen this image before?*’ appeared on screen. Participants pressed the ‘Y’/ ‘N’ keys to indicate their decision. No feedback was provided. After response, the statement *‘Rate your confidence’* appeared on screen, along with a 3-point (low, medium, high) clickable rating scale. Given our image set constraints, the rating could not be analysed using signal detection methods, hence it is not reported here (see *Supplementary Information—Complementary measures of recognition*, for further analyses where we use this data to show that we have no evidence for systematic response bias). The old/new response and confidence rating were self-paced. In contrast to the metamemory block, where a constant presentation time was chosen to standardize encoding time, the test image was kept on screen indefinitely to allow participants to interrogate their memory with no time constraints. At the end of the recognition block, participants were fully debriefed.

### Vigilance checks

The study and test blocks started with practice blocks that contained 5 arbitrary pictures from a database of flowers^51^, unrelated to the experiment. One flower picture that was shown both in the study and test practice blocks was also scheduled to randomly repeat 5 times during the real test block as a vigilance check, among Lepidoptera pictures. If participants classified the flower as ‘new’ one or more times, it was taken as evidence that they did not sufficiently engage with the task. Therefore, their data was excluded from the analyses (see *Participants*). This method is similar to the ‘vigilance repeats’^14^ used in continuous recognition memory tasks to screen out inattentive participants.

### Statistical analysis

For statistical analysis, the *R* environment was used [R Core Team, 2021]. After excluding participants who failed any vigilance checks, a total of 2,850 analysable trials were available for each of the two blocks (metamemory rating and recognition). One AP image was excluded from the analyses because of a technical error in programming the experiment. For each target image (i) we calculated recognition performance aggregated across observers. Each observation was coded as 1 for a hit or 0 for a miss, resulting in N⁽ⁱ⁾ total observations for image i, with H⁽ⁱ⁾ hits. Hit rates were then calculated as the proportion of hits per image (HR⁽ⁱ⁾ = H⁽ⁱ⁾ / N⁽ⁱ⁾), providing a measure of recognition success across the whole subject pool^14^. Note that early memorability work^14^ used the hit rate per image as a measure of recognition performance (as we do here). More recent work, however, recommends subtracting an image’s false alarm rate from the hit rate to obtain a more accurate performance estimate^16^. In our image set, lure images were only available for AP species, meaning that false alarm rates could not be calculated for both image classes. For this reason, we used hit rates in all analyses (but see Supplementary Figure S3 for data on lure images, and Supplementary Figure S5 for complementary recognition measures).

To analyse metamemory ratings and recognition responses, mixed models were fitted to the raw trial-by-trial data using the functions *lmer* and *glmer* (respectively) from the *lme4* package^52^. Mixed models allow the inclusion of individual intercepts and slopes for each subject. They are useful to account for the contribution of random variability in data due to individual differences in baseline behaviour (intercepts) and how subjects respond to experimental manipulations (slopes). This is beneficial for online studies such as ours, as viewing conditions and user focus levels cannot be strictly controlled. For the selection of mixed models we used the Akaike information criterion (AIC). ANOVA comparisons between models were conducted and those with the lowest AIC were reported in Results. Full model comparisons are included in Supplementary Tables S7 and S8. To verify model assumptions, the *check_model()* function from the *performance* R package^53^ was used. To estimate marginal and conditional R-squared values from mixed model fits, we used the *r.squaredGLMM()* function from the *MuMIn* R package^54^. Statistical significance was determined using a two-tailed significance threshold of α = 0.05. In all correlation-based analyses (metamemory-memory comparison, and consistency analysis), Spearman’s rank-order correlation (non-parametric) was used to allow for direct comparisons to results of previous image memorability research, where non-parametric correlations are most frequently reported.

To compare the relationship between metamemory ratings and recognition performance across our image set, we used the ‘think memorable/is memorable’ analysis introduced by Isola et al.^14^. We firstly calculated the average metamemory rating and hit rate for each image across participants. Subsequently, for each image, we computed the difference between metamemory rating and hit rate. Images were then ranked according to this difference to identify cases in which participants’ intuitions diverged most strongly from the observed recognition performance. Images with the largest positive differences were classified as ‘thought memorable but actually forgettable’, whereas those with the largest negative difference were classified as ‘thought forgettable but actually memorable’. To identify images for which the measures most strongly aligned, we selected images with the smallest absolute differences between metamemory rating and hit rate. Within this subset, images were classified as ‘thought memorable and memorable’ or ‘thought forgettable and forgettable’ based on whether both measures fell above or below their respective distributional thresholds (defined using the 60th and 40th percentiles). For each of these four categories, we visualised the six most extreme exemplars (shown in Fig. 3A), calculating the mean metamemory rating and hit rate across those images.

### Statistical testing of consistency measures

For the consistency analysis, as recommended by current guidelines on memorability estimation, correlations between random splits of subjects were corrected using the Spearman-Brown correction for split-half reliability^55,56^. This correction adjusts the correlation to estimate the reliability of the full dataset, compensating for the reduced data size in each split (in our case, N/2 = 25). For the split-half reliability analysis (Fig. 4A), we assessed statistical significance using a permutation test. We calculated the difference (observed Δρ) between the observed split-half reliabilities for AP and non-AP images. To test whether differences could arise by chance, we randomly permuted the AP vs. non-AP class labels 1000 times, recalculating the reliability difference between AP and non-AP (shuffled Δρ). A p-value was calculated by measuring the rate at which the shuffled Δρ exceeded the observed Δρ.

For the cumulative hit rate analysis (Fig. 4B), we compared hit rates across groups both within classes (for AP and non-AP separately) and between classes (comparing AP and non-AP). Within classes, we calculated two differences: (i) the difference between cumulative hit rates of group j and group k (independent subject splits), and (ii) the difference between group k and its shuffled baseline (chance). Between classes, we compared the extent to which the cumulative hit rate of group k exceeded the shuffled baseline for AP versus non-AP species. Because there was one additional non-AP image, the last rank (n = 29) of the non-AP condition was excluded from between-class comparisons. To assess statistical significance, we used bootstrapped confidence intervals and permutation tests. For each image rank number, the difference between cumulative hit rates across groups was computed, and 95% confidence intervals were obtained from 1,000 bootstrap resamples of the differences. This observed mean difference was then compared to a null distribution. To calculate p-values, the sign of each difference was randomly flipped (i.e., sign-flip permutation test), generating a null distribution of differences. The p-value was defined as the proportion of permuted values with equal or greater magnitude than the observed difference. Because multiple tests were conducted (one for each rank), the Bonferroni correction was used to adjust p-values, controlling for the familywise error rate.

## Supplementary Information

Supplementary Information.

## Data Availability

The data are available online from the Open Science Framework: [https://osf.io/syf2w/].
