# Supplementary material for: Natural warning signals unexpectedly shape human metamemory ratings but not image recognition success
Source: Sci Rep. 2026 Feb 25;16:10435. doi: 10.1038/s41598-026-41178-y (PMC13031780; doi:10.1038/s41598-026-41178-y)
Supplement: Supplementary file 1 — Supplementary Information. [file 41598_2026_41178_MOESM1_ESM.pdf]

Natural warning signals unexpectedly shape human metamemory ratings but not image recognition success

Federico De Filippi[1,*], **Olivier Penacchio**[2,3], **Akira R. O'Connor**[1], **Julie M. Harris**[1]

1. School of Psychology & Neuroscience, University of St Andrews, St Andrews KY16 9JP, United Kingdom
2. Bridging Research in AI and Neuroscience, Computer Vision Center, Cerdanyola del Vallès, 08193 Barcelona, Spain
3. Computer Science Department, Universitat Autònoma de Barcelona, Cerdanyola del Vallès, 08193 Barcelona, Spain

* Corresponding author (fdf1@st-andrews.ac.uk)

Supplementary information

Table of Contents

Selection of species for memory experiment **.. 2**

Conversion of hyperspectral images to sRGB colour space **........................... 2**

Influence of specimen size on behaviour **.. 6**

Complementary measures of recognition **.. 7**

Relationships between behaviour and visual metrics **................................... 9**

Statistical inference **... 10**

References **... 12**

Selection of species for memory experiment

All images implemented in the study were selected from the publicly available St Andrews database of hyperspectral images of Lepidoptera (https://arts.st-andrews.ac.uk/lepidoptera/documentation.html). For details on image acquisition, see Penacchio et al.[1] contains images of 125 species from 12 lepidopteran families: 96 aposematic (AP) and 29 non-aposematic (non-AP), sampled from museum collections located in British museums. For each image, the database also provides three metrics (luminance contrast, colour contrast, Orientation Distribution Deviation (ODD)) that characterise modelled activity in an avian visual system, according to the computational framework proposed by Penacchio et al [1]. As reported in the paper, these metrics can be used to classify butterflies as AP vs. non-AP with high accuracy. We fitted a logistic regression model to the database of neural metrics using the *R* [2] function *glm*, with luminance contrast, colour contrast, and ODD as predictors, and class (AP vs. non-AP) as the outcome variable. The logistic regression model was used to predict the overall neural signature for each image using the *R* [2] function *predict*. In other words, we used the predictive log odds that a given specimen is aposematic based on its visual statistics as a metric to select a set of stimuli that we presented to humans in the recognition memory test. When visualised in a three-dimensional pattern space (shown in *Supplementary Figure S1*) the selected stimuli occupy different regions of the space. Species that were used as memorisation targets are shown in *Supplementary Figure S2*, and lure species are shown in *Supplementary Figure S3*. We show the correlations between the visual statistics and the behavioural data in *Supplementary Figure S6*.

Conversion of hyperspectral images to sRGB colour space

The hyperspectral images from the St Andrews Hyperspectral Lepidoptera Database contain continuous information about the spectral reflectance of each pixel for several wavelengths, and as such, they required processing to be displayed as images in the conventional colour space. As proposed by Foster & Amano [3], reflectance data was converted to reflected radiance data by multiplying each pixel by the corresponding spectrum of daylight (correlated colour temperature (CCT) of 6500 K). Radiance data was subsequently converted to the RGB colour space using the CIE 1931 colour matching functions [4]. Specimens were segmented from the original hyperspectral scans and superimposed on a background with R, G, B values of 0 (‘black’).

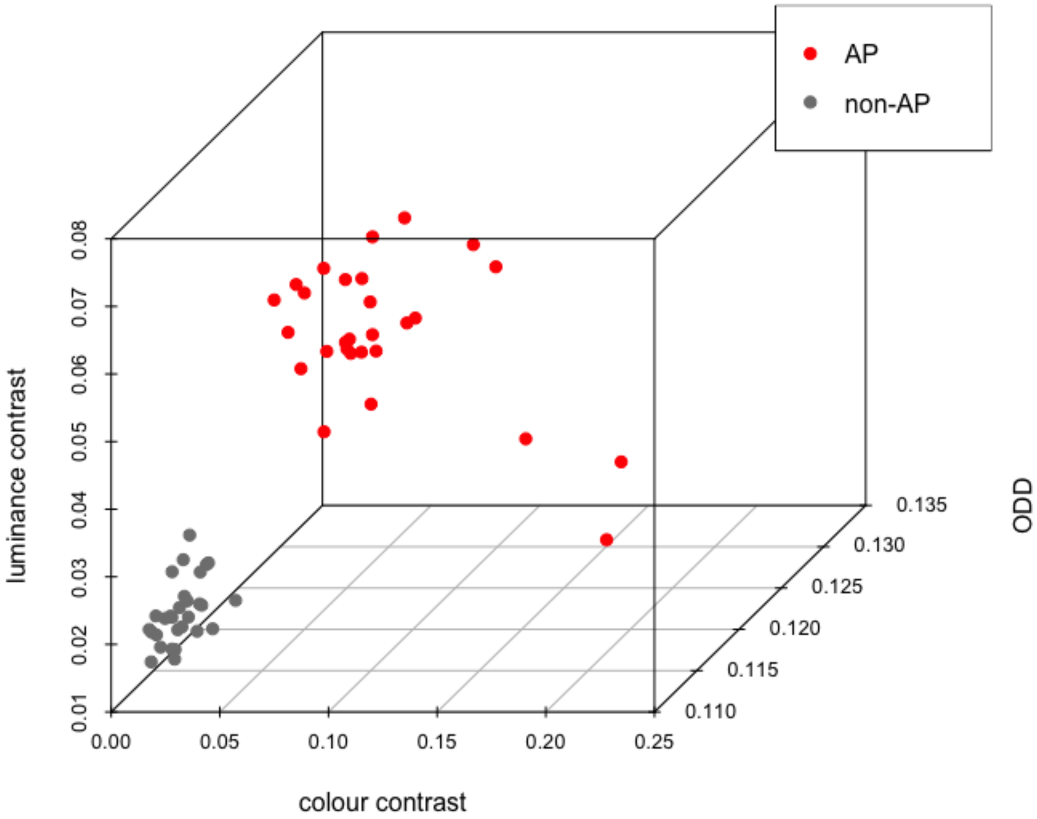

Supplementary Figure S1. Three-dimensional scatterplot of luminance contrast, colour contrast, and Orientation Distribution Departure for the Lepidoptera species (29 AP, 29 non-AP) from the St Andrews Hyperspectral Lepidoptera database [1] selected for the human memory experiment. Each point is a representative image of one species, selected based on a weighted sum of luminance contrast, colour contrast, and Orientation Distribution Deviation (ODD).

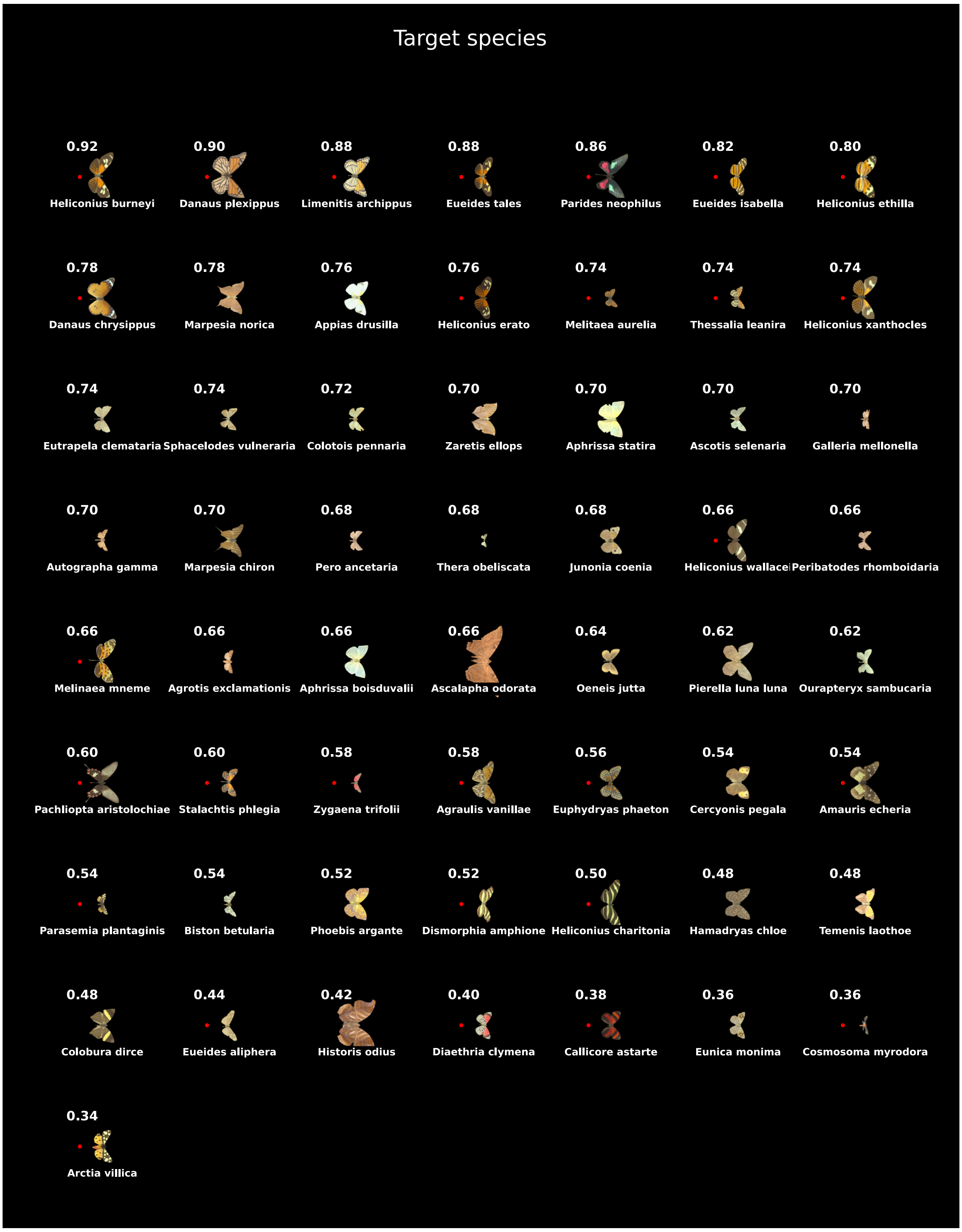

Supplementary Figure S2. Target species used in the human memory experiment, sorted by the hit rate (shown at the top-left of each species). The red dot marks aposematic species. The scientific name for each target is shown below the corresponding image.

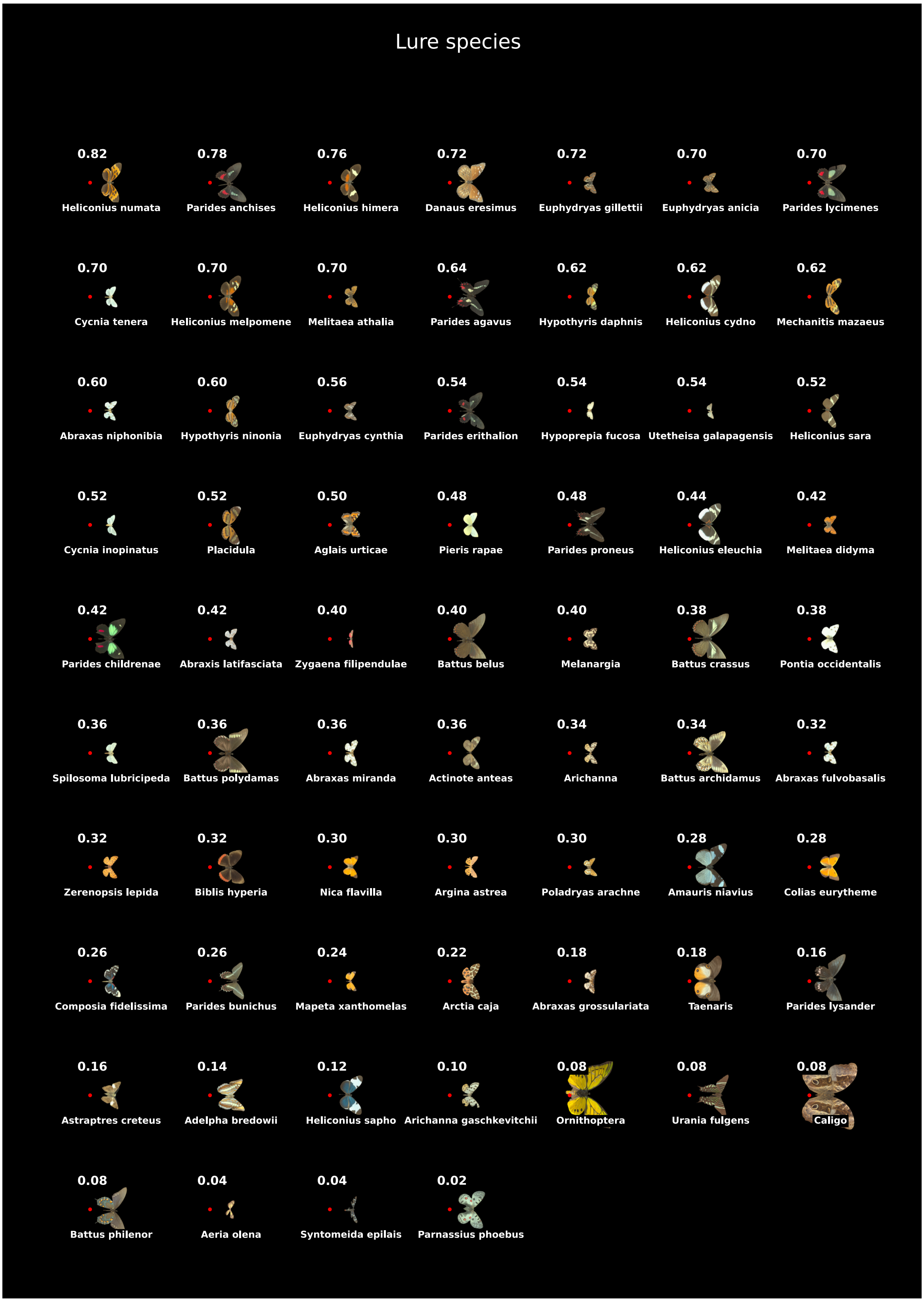

Supplementary Figure S3. Lure species used in the human memory experiment, sorted by the false alarm rate (shown at the top-left of each species). The red dot marks aposematic species. The scientific name for each lure is shown below the corresponding image.

Influence of specimen size on behaviour

As detailed above, in our stimulus set we chose to retain the natural variation in size among the selected Lepidoptera species. To assess how differences in specimen sizes influenced the experimental data, we regressed the log-transformed specimen sizes against the average metamemory ratings and hit rates. Across all images, size had a significant effect on metamemory ratings (b = 0.09, t = 4.95, SE = 0.02, $p < .001$, $R^2 = 0.23$) but not on hit rates (b = 0.03, t = 1.12, SE = 0.03, $p = .273$, $R^2 = 0.00$). This is shown by the solid lines in *Supplementary Fig. S4*. We also analysed the influence of size separately by Lepidoptera class (shown by dashed lines in *Supplementary Fig. S4*). For AP species, larger specimen size was associated with higher metamemory ratings (b = 0.12, t = 2.56, $p = .017$, $R^2 = 0.20$) and higher recognition hit rates (b = 0.06, t = 3.00, $p = .006$, $R^2 = 0.26$). For non-AP species, specimen size showed a strong positive association with metamemory ratings (b = 0.09, t = 7.34, $p < .001$, $R^2 = 0.67$) but was not significantly related to hit rate (b = −0.04, t = −1.36, $p = .186$, $R^2 = 0.06$).

This pattern of data suggests that, although size influenced metamemory ratings and recognition for AP species, it is unlikely to have facilitated recognition for non-AP species. Thus, there is no evidence that specimen size masked recognition advantages, supporting our main findings and conclusions.

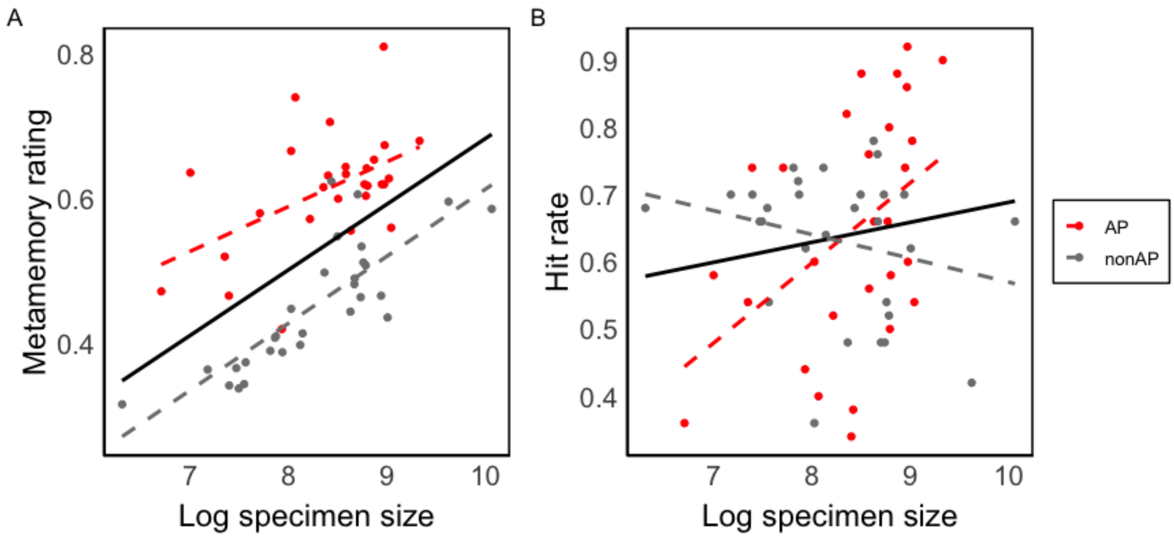

Supplementary Figure S4. Scatterplot and best linear fits of the correlations between specimen size (measured as the total number of pixels contained inside the specimen) and (**A**) metamemory rating (**B**) hit rate. Each point displays the measures for one image. Solid black lines show linear fits across all species. Dashed coloured lines show linear fits split by Lepidoptera class (AP, non-AP).

Complementary measures of recognition

In addition to the hit rates from the recognition task reported in the main body of the paper, we examined two additional measures of recognition performance: mean recognition confidence and reaction time, shown in *Supplementary Figure S5*. Analysis of confidence and reaction time can address whether our restricted choice of stimulus set could have introduced response bias.

Mean recognition confidence was analysed using a linear mixed-effects model (including random intercepts and slopes for participants) and showed a small but significant increase for aposematic (AP) relative to non-aposematic species (β = 0.08, SE = 0.04, $t(49) = 2.32$, $p = .025$; *Supplementary Figure S5A*). Reaction times were also analysed using a linear mixed-effects model and did not differ significantly between AP and non-AP species (β = −76.9 ms, SE = 159.3, $t(49) = -0.48$, $p = .631$; *Supplementary Figure S5B*).

These measures provide additional support for the conclusion that recognition performance did not significantly differ between AP and non-AP species. Confidence was not reported in the main body of the paper because the study design (as explained in *Materials & Methods*) precluded analysis using standard signal-detection approaches. However, this is a useful control measure because of our image set constraints: in the recognition test, only AP species were used as lures. If this was inferred by participants, they would be systematically more unsure when recognising AP species, but our data shows the opposite pattern (*Supplementary Figure S5A*). Note that reaction times should be interpreted cautiously, as responses were self-paced and no time limits were imposed.

Neither measure showed substantial differences between the AP and non-AP stimulus categories. Thus, there is no support for a possible effect of response bias.

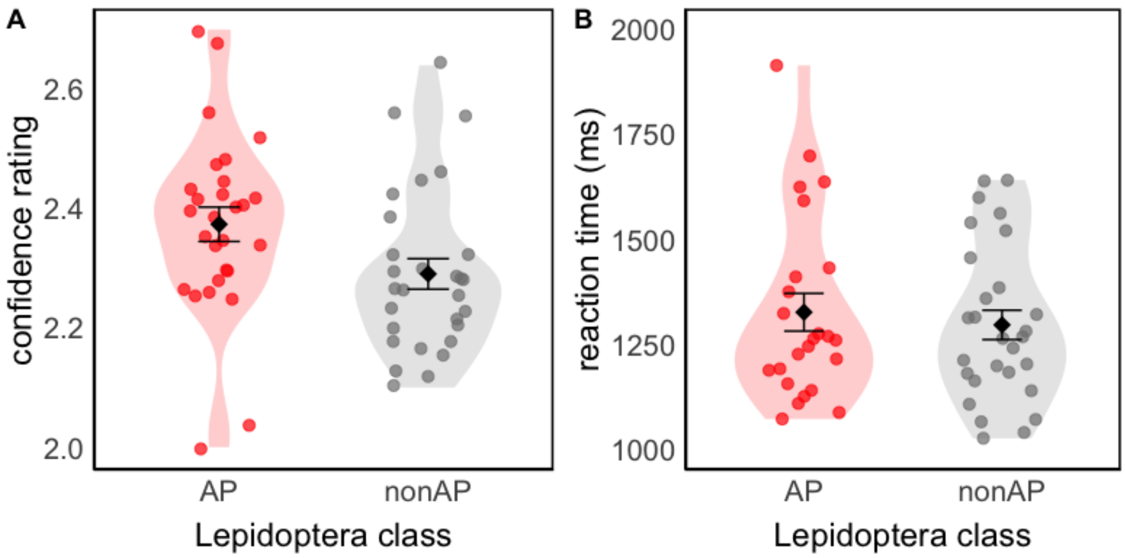

Supplementary Figure S5. (**A**): Confidence rating and (**B**) reaction time for each image, grouped by whether species were aposematic (AP, red dots) or not (non-AP, grey dots). Each point shows the mean rating of one image across the subject pool. Points are randomly jittered along the x-axis. Error bars display the mean rating ± 1 standard error of the mean.

Relationships between behaviour and visual metrics

To assess associations between the three visual statistics used by our model [1] and behavioural measures, correlations were computed between luminance contrast, colour contrast, and ODD and both metamemory ratings (*Supplementary Fig. S5A–C*) and recognition hit rate (*Supplementary Fig. S5D–F*). Across all images, metamemory ratings were significantly correlated with all three metrics (luminance contrast: r = 0.69, $p < .001$; colour contrast: r = 0.69, $p < .001$; ODD: r = 0.56, $p < .001$). Recognition hit rate was not significantly correlated with any of the metrics (luminance contrast: r = 0.06, $p = .653$; colour contrast: r = −0.05, $p = .737$; ODD: r = 0.05, $p = .698$). Class-specific trends are shown by the dashed regression lines in *Supplementary Fig. S6*.

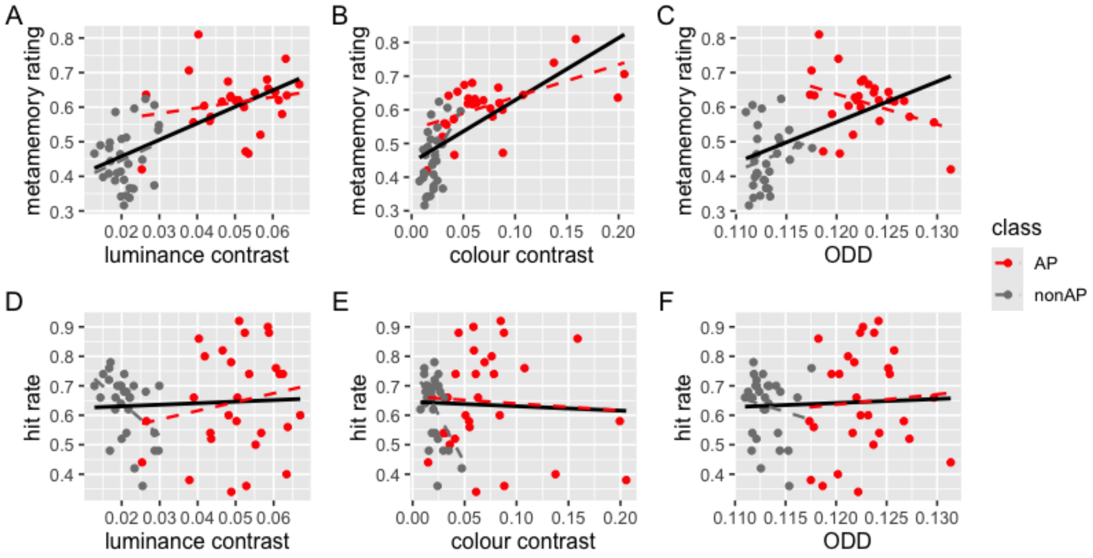

Supplementary Figure S6. Relationships between behavioural measures and visual statistics. Scatterplots show metamemory ratings (**A–C**) and recognition hit rate (**D–F**) plotted against luminance contrast, colour contrast, and Orientation Distribution Deviation (ODD). Each point corresponds to a single image and is colour-coded by Lepidoptera class. Solid black lines show the best-fitting linear regressions across all images. Coloured dashed lines show the best-fitting linear regressions split by Lepidoptera class.

Statistical inference

To identify the most appropriate mixed-effects structures to model the experimental data, we fitted a series of mixed-effects models and compared them using likelihood-ratio tests. The tables below report the model selection results. *Supplementary Table S4* reports model comparisons for metamemory ratings, and *Supplementary Table S5* reports model comparisons for recognition accuracy.

Supplementary Table S7. Likelihood-ratio model comparisons for metamemory rating models. The model highlighted in grey was reported in the paper.

Model	Effects		AIC	BIC	Log Likelihood	χ^2	df	p-value
	Fixed	Random observer ID (experimental setting)						
Model 1 (null)		Intercept	-741.62	-723.76	373.81			
Model 2	+ Lepidoptera class	Intercept	-1,220.05	-1,196.23	614.02	480.42	1	< .001
Model 3	+ Lepidoptera class	Intercept + Lepidoptera class	-1,423.05	-1,387.32	717.52	207.00	2	< .001

Fixed effects	Estimate	SE	t-value	p-value
Intercept	0.451	0.224	20.118	< .001
Lepidoptera class	0.162	0.019	8.745	< .001

Random effects	Variance	SD
Intercept	0.452	0.155
Lepidoptera class	0.150	0.122

Model fit: R^2 (marginal): 0.108; R^2 (conditional): 0.475

Supplementary Table S8. Likelihood-ratio model comparisons for recognition accuracy models. The model highlighted in grey was reported in the paper.

Model	Effects		AIC	BIC	Log Likelihood	χ^2	df	p-value
	Fixed	Random observer ID (experimental setting)						
**Model 1 (null)**		Intercept	3584.70	3596.60	-1790.40			
**Model 2**	+ Lepidoptera class	Intercept	3586.10	3603.90	-1790.00	0.658	1	0.417
**Model 3**	+ Lepidoptera class	Intercept + Lepidoptera class	3565.00	3594.80	-1777.50	25.044	2	< .001

**Fixed effects**	Estimate	SE	z-value	p-value
**Intercept**	0.602	0.123	4.883	< .001
**Lepidoptera class**	0.057	0.127	0.453	0.651

**Random effects**	Variance	SD
**Intercept**	0.585	0.765
**Lepidoptera class**	0.456	0.675

Model fit: R^2 (marginal): 0.000; R^2 (conditional): 0.108

References

1. Penacchio, O. *et al.* A computational neuroscience framework for quantifying warning signals. *Methods in Ecology and Evolution* **15**, 103–116 (2024).
2. R Foundation for Statistical Computing, R. C. T. R: A language and environment for statistical computing. (2021).
3. Foster, D. H. & Amano, K. Hyperspectral imaging in color vision research: tutorial. *J. Opt. Soc. Am. A, JOSAA* **36**, 606–627 (2019).
4. CIE JTC 2. CIE 018:2019 The Basis of Physical Photometry, 3rd Edition. doi:10.25039/TR.018.2019.
